# Isolation and Characterization of Enterocin-Producing *Enterococcus faecium* Strains from Algerian Traditional Food “Dried Figs Marinated in Olive Oil”: Functional and Safety Evaluations

**DOI:** 10.3390/foods14050766

**Published:** 2025-02-24

**Authors:** Mohamed Merzoug, Keltoum Bendida, Marwa Aireche, Zohra Yasmine Zater, Chaimaa Naila Brakna, Amaria Ilhem Hammadi, Yasmine Saidi, Svetoslav Dimitrov Todorov, Djamal Saidi

**Affiliations:** 1 Higher School of Biological Sciences of Oran, BP 1042 Saim Mohamed, Cité Emir Abdelkader (EX-INESSMO), Oran 31000, Algeria; midotech31@yahoo.fr (M.M.); keltoumbendida2001@gmail.com (K.B.); marwa.ar231@gmail.com (M.A.); chaimaa1012@gmail.com (C.N.B.); hammadiamaria267@gmail.com (A.I.H.); yasmine.saidi@gmail.com (Y.S.); djamsaidi@gmail.com (D.S.); 2 Laboratory of Biology of Microorganisms and Biotechnology, University of Oran 1 Ahmed Ben Bella, Oran 31000, Algeria; yassminezater93@gmail.com; 3 ProBacLab, Laboratório de Microbiologia de Alimentos, Departamento de Alimentos e Nutrição Experimental, Food Research Center, Faculdade de Ciências Farmacêuticas, Universidade de São Paulo, São Paulo 05508-000, SP, Brazil

**Keywords:** *Enterococcus faecium*, enterocins, genetic diversity, natural preservatives, starter cultures, clean-label foods

## Abstract

The increasing consumer demand for natural and sustainable food preservation methods has highlighted the potential of lactic acid bacteria (LAB) and their bioactive metabolites, particularly bacteriocins, as effective antimicrobial agents. This study aimed to isolate and characterize *Enterococcus faecium* strains from Algerian traditional dried figs marinated in olive oil, a nutrient-dense and underexplored food matrix. Twelve isolates were identified as *E. faecium* using MALDI-TOF MS and 16S rRNA gene sequencing, ensuring precise taxonomic classification. Genotypic analyses (BOX-PCR, GTG-PCR, and ERIC-PCR) revealed substantial genetic diversity, with BOX-PCR demonstrating superior discriminatory power. Functional screening confirmed the presence of enterocin genes, including *entA* (100% of strains), *entB* (60%), and *entL50A*/B (20%), which correlated with inhibition zones against *Enterococcus faecium* VCY, *Micrococcus luteus* GPE 3001, *Staphylococcus aureus* ATCC 25923, *Pseudomonas aeruginosa* ATCC 27853, and *Acinetobacter lwoffii* GPE 3002. Genotype–phenotype correlation analysis identified strain HFM7 as the most potent antimicrobial strain, exhibiting the largest inhibition zone (20.0 ± 1.0 mm) and harboring three enterocin genes (*entA*, *entL50A*, and *entL50B*). Protease sensitivity confirmed the proteinaceous nature of the antimicrobial compounds. Importantly, no virulence factors (*esp*, *gelE*, and *hyl*) or antibiotic resistance genes (*vanA*, *vanB*, *ermA*, *ermB*, and *aac(6′)-Ie-alph(2″)*) were detected, underscoring the safety of these isolates for food applications. These findings suggest that *E. faecium* strains from traditional foods are promising candidates as natural biopreservatives and starter cultures in clean-label food systems. By bridging traditional food ecosystems and modern biotechnological advancements, this study provides a foundation for sustainable, minimally processed food preservation strategies with potential applications in enhancing food safety and shelf life.

## 1. Introduction

The increasing demand for natural and sustainable food preservation techniques has reignited interest in lactic acid bacteria (LAB) and their bioactive metabolites, particularly bacteriocins. These antimicrobial peptides, including enterocins produced by *Enterococcus* species, demonstrate broad-spectrum activity against foodborne pathogens and spoilage microorganisms [1,2]. Their remarkable stability under diverse processing conditions further highlights their potential as effective agents for natural preservation strategies [3,4,5]. In this context, traditional fermented foods serve as valuable and underexplored reservoirs of LAB, including potential probiotic strains with notable antimicrobial properties. Integrating LAB and their metabolites into food preservation represents an innovative approach to ensuring food safety while aligning with consumer preferences for natural alternatives [6,7].

These beneficial bacteria not only inhibit foodborne pathogens but also contribute to flavor development and texture improvement, making them essential for both traditional and modern food processing. They play a multifunctional role in enhancing food safety, quality, and nutritional value, particularly in fermented foods and beverages. Their ability to produce bioactive compounds such as bacteriocins, organic acids, and exopolysaccharides underscores their significance in natural preservation strategies [8].

Furthermore, bacteriocins, including enterocins, play a crucial role in food safety and preservation. In addition to their well-established antimicrobial activity, they extend beyond traditional applications, offering novel solutions to persistent challenges in food preservation, such as controlling bacterial spores [9]. Starter cultures, in particular, play a crucial role in ensuring the safety of fermented products by suppressing undesirable microorganisms and promoting the growth of beneficial strains [10].

As a result, fermented foods have been a cornerstone of human diets for millennia, offering extended shelf life, enhanced nutritional value, and potential health benefits [11]. Modern research highlights the need to characterize microbial communities within these foods to harness their biotechnological potential while ensuring safety [12]. By acting as a bridge between cultural heritage and modern food technology, starter cultures derived from traditional fermented foods enable the global dissemination of diverse fermentation practices while improving product consistency, safety, and quality [13].

Among traditional food preparations, the combination of dried figs and olive oil is particularly noteworthy, merging the health-promoting properties of both components, both of which are central to the Mediterranean diet. Figs are a rich source of dietary fiber, essential minerals, and antioxidants, while olive oil provides monounsaturated fats and phenolic compounds with well-established cardiovascular and anti-inflammatory benefits [14]. Recent studies have further highlighted the functional potential of figs, demonstrating their bioactive compounds’ role in health management [15].

Starter cultures have revolutionized the fermentation industry by ensuring microbial consistency, enhancing sensory attributes, and suppressing undesirable microorganisms. However, the use of *Enterococcus* species as starter cultures presents both opportunities and challenges. While they contribute to food safety and quality, some strains may harbor virulence factors and antibiotic resistance genes, necessitating comprehensive genetic and functional evaluations [16]. Advances in molecular biology and genomic analysis now allow for a better distinction between commensal and potentially pathogenic *Enterococcus* strains. For instance, Oh et al. [17] isolated and characterized an *E. faecium* strain from traditional Korean fermented meju, highlighting its potential as a safe starter culture. *Enterococcus* spp. have also been reported in fermented foods such as boza [18,19], olives [20,21], cheeses [22,23,24], salami [25,26], and fermented vegetables [27].

In Algeria, traditional foods play an integral role in cultural and dietary practices, yet their microbial ecosystems remain underexplored. Dried figs marinated in olive oil represent a nutrient-rich preparation enriched with antioxidants, polyphenols, and beneficial fatty acids, providing an untapped resource for isolating antimicrobial-producing strains, including enterocin-producing *Enterococcus* species [28].

The present study aimed to isolate and characterize enterocin-producing *Enterococcus* strains from dried figs marinated in olive oil, examining their genotypic diversity alongside their antimicrobial potential and safety to better understand their role in food preservation. Through phenotypic, genotypic, and functional analyses, this research explored their utility in natural food preservation while addressing the risks associated with their virulence and resistance traits. The strains were screened against clinical and foodborne pathogens to assess their antimicrobial potential. By leveraging the biotechnological potential of these strains and their application as starter cultures, this study supports the development of “clean label” products that are natural, minimally processed, and culturally significant, contributing to the advancement of sustainable food preservation techniques [13].

## 2. Materials and Methods

### 2.1. Strains Isolation and Growth Conditions

To isolate bacterial strains, 25 g of a six-month-old homemade preparation of dried figs marinated in olive oil were homogenized with 225 mL of sterile physiological saline solution (0.85%, *w*/*v*, NaCl) using a stomacher (400 Circulator Lab Blender, Seward, West Sussex, UK) for 2 min. Serial tenfold dilutions (10^−1^ to 10^−8^) were performed to decrease microbial density and ensure the isolation of single colonies on plates. Aliquots (100 μL) from appropriate dilutions were plated in duplicate on de Man, Rogosa, Sharpe (MRS) agar (Fluka, St. Louis, MO, USA) for the isolation of LAB. Plates were incubated under aerobic conditions at 30 °C for 24–72 h to allow colony development. Colonies with distinct morphology were selected on the basis of phenotypic variation and were further purified using the quadrant streak method on MRS supplemented with 2% agar (Fluka). The purified isolates were subjected to phenotypic characterization, which included Gram staining, catalase activity, bile-esculin hydrolysis, tolerance to 6.5% NaCl, and morphological examination under light microscopy [29]. Only isolates identified as Gram-positive, catalase-negative cocci were retained for subsequent screening as potential enterocin-producing enterococci.

For preservation, isolates were stored using two complementary methods: freeze-drying for long-term storage at room temperature and freezing at −80 °C in liquid MRS broth supplemented with 50% (*v*/*v*) glycerol to ensure viability and genetic stability over extended periods [30].

### 2.2. Bacterial Isolate Identification

#### 2.2.1. Identification of Selected Isolates by MALDI Biotyper Sirius GP System

Bacterial isolates were identified using the MALDI Biotyper Sirius GP system (Bruker Daltonics GmbH, Bremen, Germany) at the Genomics Technology Platform of the Higher School of Biological Sciences of Oran (ESSBO, Oran, Algeria). Isolates were cultured as described before on MRS agar at 30 °C for 24 h. Individual colonies were transferred to an MBT Biotarget 96 plate (Bruker) and treated with 1 μL of 70% formic acid to facilitate cell lysis and protein extraction. Subsequently, 1 μL of alpha-cyano-4-hydroxycinnamic acid (HCCA) matrix solution was applied to each sample [31]. This preparation method adheres to the standard protocols for bacterial identification recommended in the Bruker Daltonics Manual (https://www.bruker.com, accessed on 14 October 2024). Mass spectra were analyzed using FlexControl software (version 3.4, Bruker) and MBT Compass HT software (version 5.1, Bruker), with calibration performed using the Bacterial Test Standard (BTS), which contains *Escherichia coli* extracts with RNase A and myoglobin. This approach allows for the reliable identification of bacterial species based on their unique proteomic fingerprints [32].

#### 2.2.2. Molecular Identification

##### 16S rRNA Amplification with Colony PCR

The 16S rRNA gene, a conserved marker for bacterial identification, was amplified using Colony PCR (polymerase chain reaction) directly from bacterial cells as the template, without prior DNA extraction or purification [33]. Universal primers 16S Forward 8F and 16S Reverse 1492R (Table 1), synthesized at the Genomics Technology Platform of the Higher School of Biological Sciences of Oran (ESSBO), were used for amplification. PCR reactions were prepared in a final volume of 50 µL using 2× PCR Master Mix (Thermo Scientific, Waltham, MA, USA) and performed on the SimpliAmp thermal cycler (Applied Biosystems, Foster City, CA, USA). Thermal cycling conditions consisted of an initial denaturation at 94 °C for 10 min, followed by 35 cycles of denaturation at 94 °C for 45 s, annealing at 54 °C for 45 s, and extension at 72 °C for 90 s, with a final extension at 72 °C for 10 min to ensure complete elongation. Amplicons (~1500 base pairs) were verified using 1% agarose gel electrophoresis in TBE buffer, alongside GeneRuler Express DNA Ladder (Thermo Scientific) for size estimation.

##### Sanger Sequencing Analysis

Prior to sequencing, PCR products were purified using ExoSAP-IT (Applied Biosystems) following the manufacturer’s instructions to remove residual primers and dNTPs [34,35]. Sanger sequencing was conducted with the BigDye Terminator v3.1 Cycle Sequencing Kit (Applied Biosystems). Each 10 µL sequencing reaction included 1 µL of BigDye Ready Reaction Mix, 2 µL of 5× Sequencing Buffer, 1 µL of primer (forward or reverse, 3.2 pmol/µL), 1 µL of purified PCR product (10–40 ng) and 5 µL of nuclease-free water. The thermal cycling conditions consisted of 25 cycles of denaturation at 96 °C for 10 s, annealing at 50 °C for 5 s, and extension at 60 °C for 4 min. Sequencing reaction products were purified using the DynaBeads Sequencing Clean-Up Kit (Thermo Scientific) to effectively remove excess dye terminators and contaminants. The purified products were eluted in Hi-Di Formamide (Thermo Scientific) and analyzed via capillary electrophoresis using the 3500 Genetic Analyzer (Thermo Scientific) at the Genomics Technology Platform of the Higher School of Biological Sciences of Oran (ESSBO).

##### Phylogenetic and Taxonomic Analysis of 16S rRNA Sequences

Chromatograms generated from sequencing were analyzed using SnapGene Viewer (version 6.0.2, Insightful Science, Boston, MA, USA) and ChromasPro (version 2.1.8, Technelysium Pty Ltd., Brisbane, Australia) to evaluate sequence quality and identify potential errors. The obtained sequences were aligned and compared against the NCBI GenBank database using the online Basic Local Alignment Search Tool BLASTn to determine the closest taxonomic matches [36]. Subsequently, the 16S rRNA gene sequences of the isolates were aligned using the ClustalW algorithm [37]. A phylogenetic tree was constructed in MEGA11 software (version 11.0.13, Center for Evolutionary Medicine and Informatics, Biodesign Institute, Tempe, AZ, USA) [38] using the neighbor-joining (NJ) method with 1000 bootstrap replications to assess the robustness of the inferred evolutionary relationships [39]. To ensure accurate taxonomic classification, a high sequence similarity threshold (>99%) was used to confirm the identity of bacterial strains, facilitating reliable identification of *E. faecium* and its phylogenetic context.

**Table 1:** Primers used in this study.

Category	Target Gene	Abbreviation	Forward Primer (5′ → 3′)	Reverse Primer (5′ → 3′)	Amplicon Size (bp)	Tm	References
**Molecular identification**	16S rRNA	16S rRNA	AGAGTTTGATCCTGGCTCAG	ACGGCTACCTTGTTACGACTT	1485	56	[40]
**Genotyping**	ERIC	ERIC	ATGTAAGCTCCTGGGGATTCAC	AAGTAAGTGACTGGGGTGAGCG	/	52	[41,42]
	(GTG)_5_	(GTG)_5_	GTGGTGGTGGTGGTG		/	45	[43,44]
	BOX-PCR	BOX-PCR	CTACGGCAAGGCGACGCTGACG		/	53	[44,45]
**Enterocin genes**	Enterocin A	*entA*	GGTACCACTCATAGTGGAAA	CCCTGGAATTGCTCCACCTAA	138	55	[46]
	Enterocin B	*entB*	CAAAATGTAAAAGAATTAAGTACG	AGAGTATACATTTGCTAACCC	201	56	[46]
	Enterocin L50A	*entL50A*	ATGGGAGCAATCGCAAAATTA	TTTGTTAATTGCCCATCCTTC	135	56	[47]
	Enterocin L50B	*entL50B*	ATGGGAGCAATCGCAAAATTA	TAGCCATTTTTCAATTTGATC	274	58	[46]
	Enterocin P	*entP*	ATGAGAAAAAAATTATTTAGTTT	TTAATGTCCCATACCTGCCAAACCAG	216	41	[48]
	Enterocin Q	*entQ*	GGAATAAGAGTAGTAGTGGAATACTGATATGAGAC	AAAGACTGCTCTTCCGAGCAGCC	653	60	[48]
	Enterocin AS-48	*entAS-48*	GAGGAGTATCATGGTTAAAGA	ATATTGTTAAATTACCAA	339	56	[48]
	Enterocin 31	*ent31*	CCTACGTATTACGGAAATGGT	GCCATGTTGTACCCAACCATT	130	58	[48]
	Cytolysin	*cyl*	GGCGGTATTTTTACTGGAGT	CCTACTCCTAAGCCTATGGTA	248	56	[47]
	Enterocin CRL35	*entCRL35*	GCAAACCGATAAGAATGTGGGAT	TATACATTGTCCCCACAACC	490	55	[49]
	Mundticin KS	*munKS*	TGAGAGAAGGTTTAAGTTTTGAAGAA	TCCACTGAAATCCATGAATGA	380	55	[49]
**Virulence factors**	Enterococcal surface protein	*esp*	ACGTGGATGTAGAGTTTGC	GAATATGTCACTACAACCGTAC	5791	57	[50]
	Gelatinase E	*gelE*	ACCCCGTATCATTGGTTT	ACGCATTGCTTTTCCATC	419	54	[51]
	Hyaluronidase	*hyl*	ACAGAAGAGCTGCAGGAAATG	GACTGACGTCCAAGTTTCCAA	276	55	[42]
**Antibiotic resistance genes**	Vancomycin A	*vanA*	CATGAATAGAATAAAAGTTGCAATA	CCCCTTTAACGCTAATACGATCAA	1030	58	[52]
	Vancomycin B	*vanB*	GTGACAAACCGGAGGCGAGGA	CCGCCATCCTCCTGGAAAAAA	433	64	[52]
	Erythromycin Ribosomal Methylase A	*ermA*	TCTAAAAAGCATGTAAAAGAA	TGATTATAATTATTTGATAGCTTC	645	50	[53]
	Erythromycin Ribosomal Methylase B	*ermB*	GAAAAGGTACTCAACCAAATA	AGTAACGGTACTTAAATTGTTTAC	639	54	[53]
	Aminoglycosidase Acetyltransferase (6′)-Aminoglycoside Phosphotransferase (2″)	*aac(6′)-Ie-alph(2″)*	CCAAGAGCAATAAGGGCATA	CACTATCATAACCACTACCG	220	44	[54]

#### 2.2.3. Genotyping and Molecular Profiling Using REP-PCR

To ensure genetic distinctiveness and eliminate duplicate isolates of *Enterococcus* strains, genotyping was conducted using repetitive sequence-based PCR (REP-PCR) with GTG, BOX, and ERIC primers [41,42,43,44,45]. This method provides high-resolution differentiation among strains, supporting robust downstream analyses [55,56,57]. Genomic DNA was extracted using the PureLink Genomic DNA Mini Kit (Thermo Scientific), supplemented with 10 mg/mL lysozyme for efficient cell lysis [58]. DNA quality was confirmed using the ScanDrop spectrophotometer (Analytik Jena, Jena, Germany), yielding A260/A280 ratios of 1.8–2.0 and A260/A230 ratios > 2.0, while gel electrophoresis verified DNA integrity. PCR reactions (30 µL) were prepared with 2X PCR Master Mix (Thermo Scientific), 3 µL of primer (10 µM), 1.5 µL bovine serum albumin (BSA; 20 mg/mL), 1 µL genomic DNA (~50 ng), and nuclease-free water. Amplifications were performed on a SimpliAmp Thermal Cycler (Applied Biosystems) under primer-specific thermal conditions, including denaturation, annealing, and elongation steps. The resulting PCR products were separated on a 1.5% (*w*/*v*) agarose gel in 1× TAE buffer at 80 V for 120 min. DNA bands were visualized using a ChemiDoc Imaging System (Bio-Rad Laboratories, Hercules, CA, USA) after staining with SYBR Safe DNA Gel Stain (Thermo Scientific). A GeneRuler Express DNA Ladder (Thermo Scientific) served as the molecular size marker. Electrophoretic patterns were analyzed using GelJ software (version 2.0, SourceForge Headquarters, San Diego, CA, USA) [59] to normalize migrations relative to the molecular weight marker. Genetic distances were calculated using Dice and Jaccard similarity coefficients, and dendrograms were constructed using the UPGMA clustering method to reveal relationships among strains.

### 2.3. Bacteriocinogenic Strain Screening and Assays

Twelve bacterial isolates were assessed for their antimicrobial properties using a well-diffusion assay [60]. The indicator strain, *E. faecium* VCY, was isolated from cow’s milk collected in the Misserghin rural forest (Oran, Algeria). The other strains, *Micrococcus luteus* GPE 3001, *Acinetobacter lwoffii* GPE 3002, *Pseudomonas aeruginosa* ATCC 27853, and *Staphylococcus aureus* ATCC 25923, were selected from the genomics technology platform collection (ESSBO). Fresh 18 h cultures of the test strains were standardized to a 0.5 McFarland standard (approximately 1.5 × 10^8^ CFU/mL) and inoculated onto soft MRS and Luria–Bertani (LB) agar plates (1% *w*/*v* agar) (Merck, Darmstadt, Germany) to create a uniform layer. Wells with a 6 mm diameter were prepared using a sterile Cork borer and filled with 100 µL of cell-free supernatants obtained by centrifuging the cultures of the test strains (10,000× *g*, 15 min, 4 °C). To eliminate organic acid effects, the pH was adjusted to 6.5 using 1 M NaOH. After allowing diffusion at 4 °C for 2 h, the plates were incubated at 30 °C for 24 h under aerobic conditions. The protease sensitivity of the antimicrobial compounds was evaluated by treating the supernatants with trypsin (1 mg/mL, (Sigma-Aldrich, Saint Louis, MO, USA) at 37 °C for 2 h [61].

The inhibition zones were measured in millimeters (mm) using a ruler. Petri dishes from the assay were imaged using a ChemiDoc Imaging System (Bio-Rad). Strains showing no detectable inhibition were assigned a value of 0 mm. All assays were performed in triplicate (n = 3) under standardized conditions.

### 2.4. Molecular Screening of Enterocin Genes, Virulence Factors, and Antibiotic Resistance

To evaluate the antimicrobial potential, virulence, and safety of *Enterococcus* strains, a PCR-based screening was performed to detect enterocin genes, virulence factors, and antibiotic resistance genes. Specific primers targeting these genes (Table 1) were synthesized at the Genomics Technology Platform of the Higher School of Biological Sciences of Oran (ESSBO). Each PCR reaction was prepared in a 25 µL volume containing 12.5 µL of 2× PCR Master Mix (Thermo Scientific), 1 µL of each primer (10 µM), 1 µL of genomic DNA (~50 ng), and nuclease-free water. The amplification protocol included an initial denaturation step at 94 °C for 3 min, followed by 35 cycles of denaturation at 94 °C for 30 s, annealing at primer-specific temperatures, and extension at 72 °C for 30–60 s (according to specifications for each set of primers). A final extension at 72 °C for 5 min ensured complete elongation. After amplification, residual primers and dNTPs were removed using ExoSAP (Thermo Scientific) to generate purified amplicons suitable for analysis. The resulting PCR products were separated on a 2% agarose gel in 1× TBE buffer, alongside the GeneRuler 100 bp DNA Ladder Mix (Thermo Scientific) serving as a molecular size marker. DNA bands were visualized using a ChemiDoc Imaging System (Bio-Rad) after staining with SYBR Safe DNA Gel Stain (Thermo Scientific). The presence of target genes was detected and analyzed computationally using Python (Version 3.10, Python Software Foundation, Wilmington, DE, USA) and the Matplotlib library (Version 3.7.1, Matplotlib Development Team, Python Software Foundation, USA).

### 2.5. Statistical Analyses

The results were reported as the mean ± standard deviation (SD). Statistical analyses were conducted using GraphPad Prism (version 9.0.0, GraphPad Software, San Diego, CA, USA). Differences among experimental groups were evaluated using one-way analysis of variance (ANOVA) and Tukey’s post-hoc test [62]. For all tests, *p* ≤ 0.001 indicates a statistically significant difference.

## 3. Results

### 3.1. Lactic Acid Bacteria Isolation

A total of twelve isolates were obtained from a six-month-old homemade preparation of dried figs marinated in olive oil. All isolates were identified as Gram-positive and catalase-negative cocci, typical of LAB. Their coccus shape was confirmed through morphological examination by light microscopy. These strains also demonstrated the ability to hydrolyze bile-esculin and thrive in 6.5% NaCl, a hallmark trait of *Enterococcus* species. When tested under various environmental conditions, the isolates exhibited robust growth across a pH range of 4.6 to 9.9 and were able to survive and proliferate at temperatures ranging from 10 °C to 45 °C.

### 3.2. Identification of Enterococcus Strains

To ensure high accuracy and reliability in species determination, the identification of bacterial isolates was performed using a combination of MALDI-TOF MS and 16S rRNA gene sequencing, integrating phenotypic and genotypic approaches.

#### 3.2.1. MALDI-TOF MS Analysis

**Figure 1:**
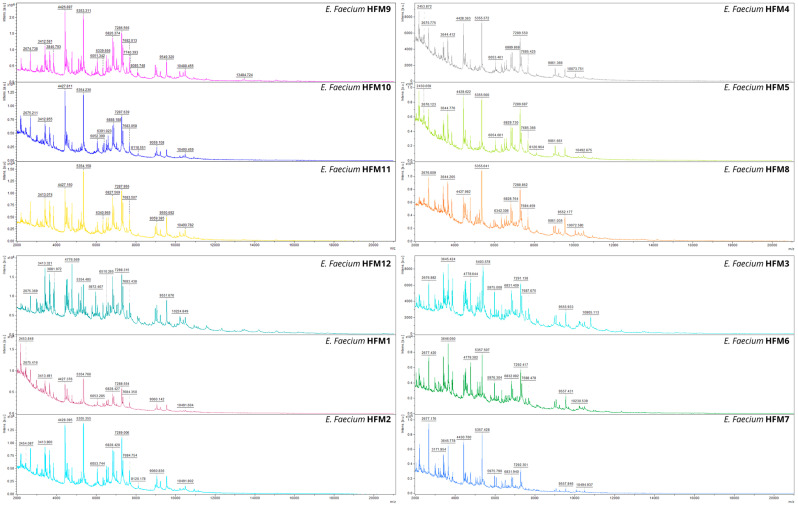
MALDI-TOF MS spectra of 12 *Enterococcus faecium* strains, illustrating characteristic mass profiles within the 2000 to 10,000 Da range. Specific peaks in the spectra highlight strain-dependent variations and conserved features among the strains.

MALDI-TOF MS identified all isolates as *E. faecium*, with log(score) values ranging from 2.30 to 2.41, confirming reliable and reproducible species identification. The Bacterial Test Standard (BTS) consistently yielded log(score) values above 2.0, further validating the system’s accuracy. Mass spectrometry analysis revealed distinct profiles for the *E. faecium* strains within the 2000–10,000 Da range (Figure 1). Peaks were consistently observed in the 2500–4000 Da, 5000–7000 Da, and 6000–8000 Da ranges, with noticeable variations in intensity across strains. Strains HFM9, HFM4, and HFM2 exhibited increased peak intensities in the 6000–8000 Da range, while strains HFM1 and HFM7 displayed lower intensities and less defined peaks. Stable peaks were observed at 4428–4778 Da, 5355–5357 Da, and 7683–7688 Da, suggesting conserved elements among the strains. Distinct evolutionary relationships were indicated by unique peaks in the 6000–8000 Da range, particularly at 7288 Da and 7685 Da. In the higher-mass range (9000–10,000 Da), significant inter-strain variation was evident, such as a unique peak at 10,805 Da found exclusively in strain HFM3, absent in the profiles of all other strains. These findings highlight both shared and strain-specific features in the mass spectrometry profiles of *E. faecium*.

#### 3.2.2. 16S rRNA Gene Sequencing

**Figure 2:**
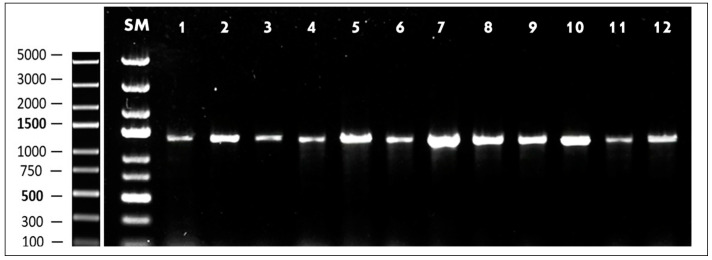
Visualization of 16S rRNA colony-PCR amplicons on a 1% agarose gel. MT: DNA size marker (GeneRuler Express DNA Ladder). Lanes: 1—HFM 1; 2—HFM 2; 3—HFM 3; 4—HFM 4; 5—HFM 5; 6—HFM 6; 7—HFM 7; 8—HFM 8; 9—HFM 9; 10—HFM 10; 11—HFM 11; 12—HFM 12.

To validate the MALDI-TOF MS results, 16S rRNA gene sequencing was performed. Colony-PCR amplification of the 16S rRNA gene generated distinct amplicons of approximately 1500 base pairs for all isolates, which were visualized as clear, single bands on 1.5% agarose gel electrophoresis stained with SYBR Safe DNA Gel Stain (Figure 2). Sequencing results confirmed that all isolates were *E. faecium*, with sequence similarities exceeding 99%. Alignment with reference strains in the NCBI GenBank database provided similarity percentages ranging from 99.1% to 100%.

#### 3.2.3. Phylogenetic Analysis

**Figure 3:**
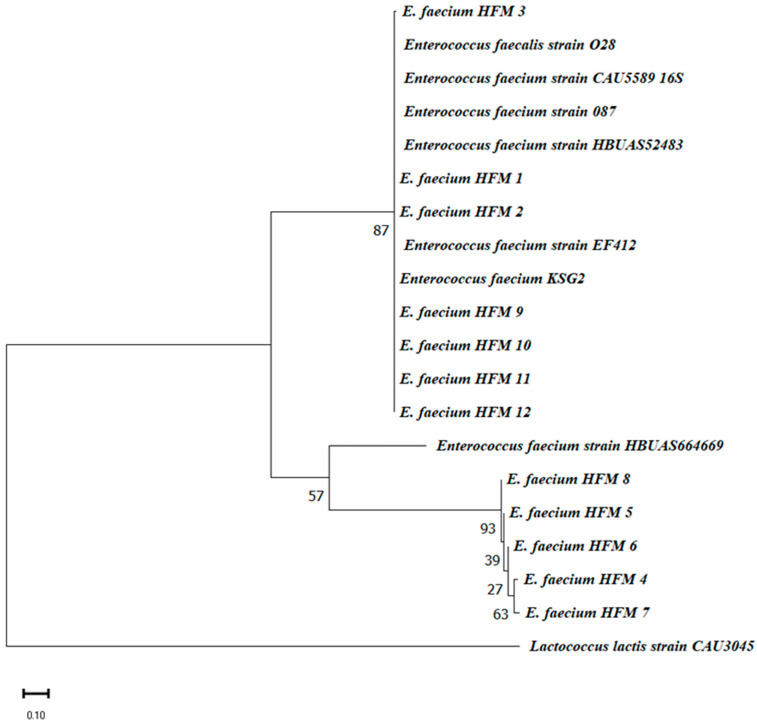
Phylogenetic tree of 12 *E. faecium* strains constructed using MEGA 11. The tree was generated on the basis of 16S rRNA gene sequences using the neighbor-joining method, with bootstrap analysis (1000 replicates) to assess branch reliability. Only bootstrap values > 35% are displayed at the nodes. The scale bar represents 10% divergence. The phylogenetic relationships demonstrate the genetic relatedness among the isolates, further confirming their classification as *E. faecium*.

Phylogenetic analysis further confirmed that all HFM isolates belong to the *E. faecium* species, aligning with the MALDI-TOF MS results. Isolates HFM 1, 2, 3, 9, 10, 11, and 12 clustered closely with reference strains EF412 and KSG2, indicating high genetic similarity. In contrast, HFM 8 formed a separate subgroup, suggesting slight genetic divergence. Isolates HFM 4, 5, 6, and 7 constituted a distinct sub-cluster, reflecting potential strain-level diversity. All isolates were clearly differentiated from the *Lactococcus lactis* outgroup, validating their classification within the *Enterococcus* genus (Figure 3).

The strong concordance between MALDI-TOF MS and 16S rRNA sequencing highlights the complementarity of these methods. MALDI-TOF MS provides rapid phenotypic identification, while 16S rRNA sequencing ensures precise genotypic validation. Combined with genotyping, these approaches establish a robust framework for bacterial taxonomy, enabling accurate and confident species identification.

#### 3.2.4. Genotyping Analysis

**Figure 4:**
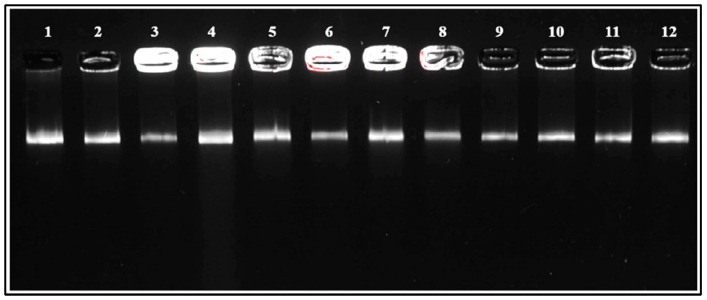
Visualization of genomic DNA on a 1% agarose gel. Lanes: 1—HFM 1; 2—HFM 2; 3—HFM 3; 4—HFM 4; 5—HFM 5; 6—HFM 6; 7—HFM 7; 8—HFM 8; 9—HFM 9; 10—HFM 10; 11—HFM 11; 12—HFM 12.

To ensure precise species identification and resolve ambiguities, a comprehensive genotyping analysis was conducted. This multi-method framework combined phenotypic (MALDI-TOF MS) and genotypic (16S rRNA sequencing) approaches with REP-PCR genotyping. The analysis confirmed species assignments, verified the genetic uniqueness of isolates, and established a robust strategy for distinguishing closely related *E. faecium* strains. High-quality genomic DNA was extracted from all isolates, as evidenced by clear, high-molecular-weight bands observed on a 1% agarose gel (Figure 4), confirming DNA integrity and suitability for downstream PCR and genotyping analyses.

**Figure 5:**
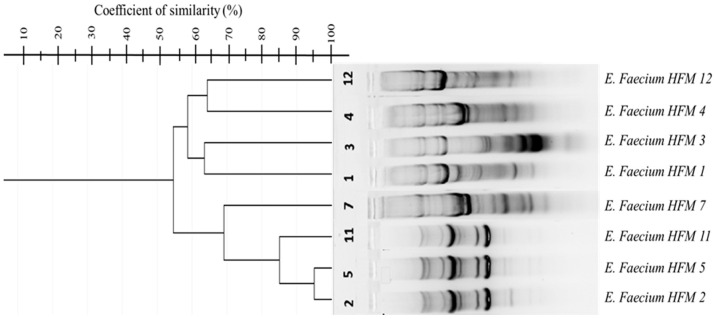
Dendrogram of GTG-PCR showing percentage similarity among eight *Enterococcus faecium* strains, calculated using the Dice similarity coefficient and clustered with the UPGMA algorithm in GelJ (version 2.0).

**Figure 6:**
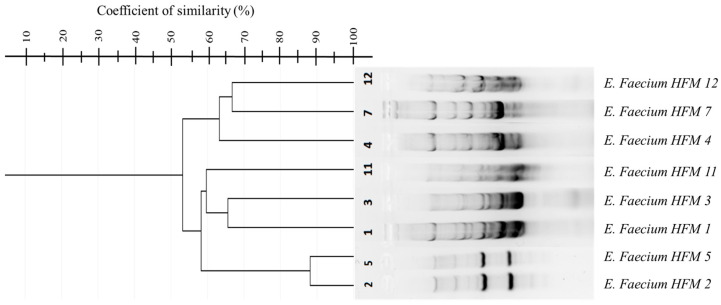
Dendrogram of BOX-PCR showing percentage similarity among eight *Enterococcus faecium* strains, calculated using the Dice similarity coefficient and clustered with the UPGMA algorithm in GelJ (version 2.0).

Three repetitive element-based PCR methods—GTG-PCR, BOX-PCR, and ERIC-PCR—were employed for genotyping. GTG-PCR and BOX-PCR demonstrated significant genetic heterogeneity among the isolates, with BOX-PCR exhibiting the highest discriminatory power (Figure 5 and Figure 6). Dendrograms constructed using the UPGMA algorithm in GelJ software (version 2.0) quantified genetic relationships [59]. Dice similarity coefficients revealed distinct clustering patterns, with strains HFM 7, HFM 11, and HFM 5 showing approximately 90% similarity, while strain HFM 2 emerged as a distinct outlier with ~50% similarity. These findings reflect unique genetic characteristics potentially shaped by ecological or evolutionary pressures.

BOX-PCR was particularly effective in resolving inter-cluster genetic diversity, especially for genetically distant strains such as HFM 2. For example, strains HFM 1 and HFM 3 displayed a high degree of similarity, suggesting a common origin or ecological niche. This observed genetic variability highlights the adaptability and ecological flexibility of *E. faecium* in complex environments such as marinated dried figs. In contrast, ERIC-PCR yielded inconsistent results due to the low abundance or uneven distribution of intergenic repetitive sequences in the bacterial genome. Despite repeated optimization attempts, its resolution was limited.

### 3.3. Screening of Bacteriocinogenic Strains and Bacteriocin-like Activity Validation

**Figure 7:**
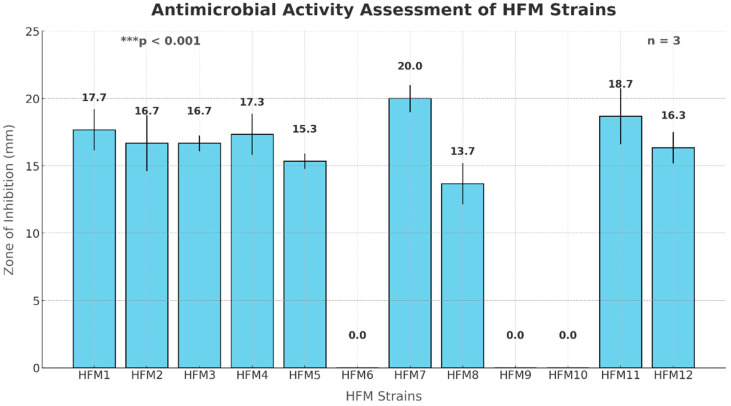
Screening and quantitative assessment of bacteriocin-like activity of HFM strains against *E. faecium* VCY using zone of inhibition analysis (n = 3, *** *p* < 0.001).

**Figure 8:**
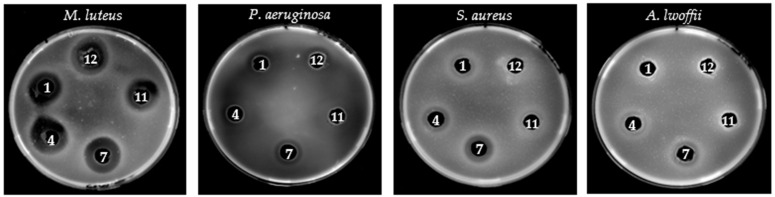
Antimicrobial activity of five selected *E. faecium* strains against four clinically relevant bacterial pathogens (*M. luteus* GPE 3001, *P. aeruginosa* ATCC 27853, *S. aureus* ATCC 25923, and *A. lwoffii* GPE 3002), evaluated using the well diffusion method [54,55]. The assay was performed on soft LB agar plates (1% *w*/*v*). Inhibition zones are shown for the following strains: (1) HFM1, (4) HFM4, (7) HFM7, (11) HFM11, and (12) HFM12.

The bacteriocinogenic potential of twelve *E. faecium* strains was assessed through auto-inhibition assays using *E. faecium* VCY as an indicator strain. Quantitative inhibition zone analysis revealed that nine strains exhibited significant antimicrobial activity (*p* < 0.001), with inhibition diameters ranging from 13.7 ± 1.5 mm to 20.0 ± 1.0 mm. The highest inhibitory effect was observed in strain HFM7 (20.0 ± 1.0 mm, *p* < 0.001), followed by HFM11 (18.7 ± 2.1 mm) and HFM4 (17.3 ± 1.5 mm). Moderate inhibition was recorded for strains HFM1, HFM2, HFM3, HFM5, HFM8, and HFM12, whereas strains HFM6, HFM9, and HFM10 displayed no detectable antimicrobial activity (Figure 7). Following the auto-inhibition assay, five strains were selected on the basis of their antimicrobial potential and tested against four bacterial pathogens, including clinically relevant species and foodborne pathogens, *M. luteus* GPE 3001 (Gram-positive), *P. aeruginosa* ATCC 27853 (Gram-negative), *S. aureus* ATCC 25923 (Gram-positive), and *A. lwoffii* GPE 3002 (Gram-negative). The results demonstrated a strain- and pathogen-dependent inhibitory activity. All selected strains exhibited strong antimicrobial activity (*p* < 0.001) against *M. luteus* GPE 3001, with inhibition zones ranging from 17 to 21 mm. Against *P. aeruginosa*, four strains showed inhibitory effects (13–17 mm), whereas strain 11 remained inactive. In the case of *S. aureus* ATCC 25923, moderate inhibition (12–15 mm) was observed for four strains, while strain 12 exhibited no detectable activity. Lastly, only strains 4 and 7 effectively inhibited *A. lwoffii* GPE 3002, with inhibition diameters of 15 and 16 mm, respectively, while the remaining three strains had no impact on its growth (Figure 8).

To confirm the proteinaceous nature of the antimicrobial compounds, proteolytic digestion with trypsin (1 mg/mL, 37 °C, 2 h) was conducted under identical conditions, leading to a complete loss of inhibition zones in all active strains. This suggests the production of bacteriocin-like inhibitory substances (BLIS). The reproducibility of these findings was validated through three independent experiments (n = 3). Statistical analysis using one-way ANOVA followed by Tukey’s post-hoc test confirmed significant variations in antimicrobial activity among the active strains (F = 24.53, df = 11, *p* < 0.001).

### 3.4. Screening of Enterocin Genes, Virulence Factors, and Antibiotic Resistance Genes

A functional screening of *E. faecium* strains was conducted to evaluate their antimicrobial potential and safety. PCR analysis targeted 19 genes, comprising 11 enterocin genes, 3 virulence genes, and 5 antibiotic resistance genes. The results revealed the presence of specific enterocin genes, while no virulence or antibiotic resistance genes were detected, confirming the safety of these strains for food-related applications.

**Figure 9:**
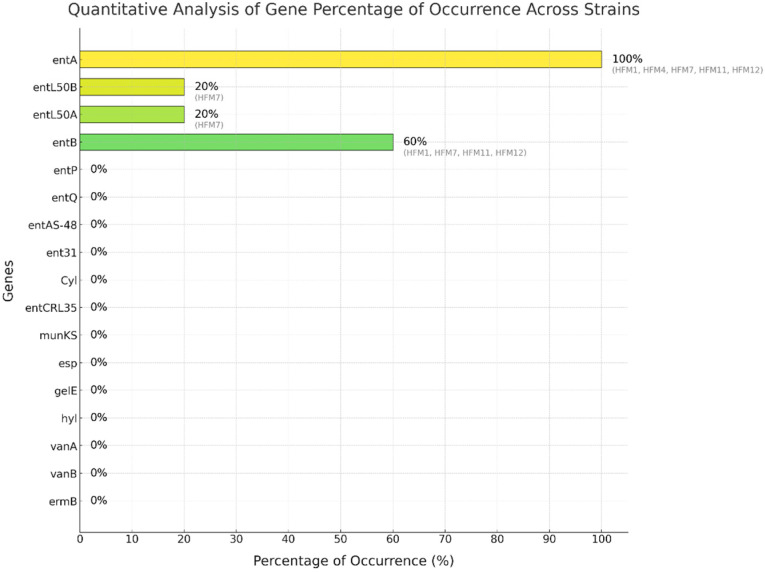
Quantitative analysis of gene occurrence across strains and the presence of enterocin, virulence, and antibiotic resistance genes.

**Figure 10:**
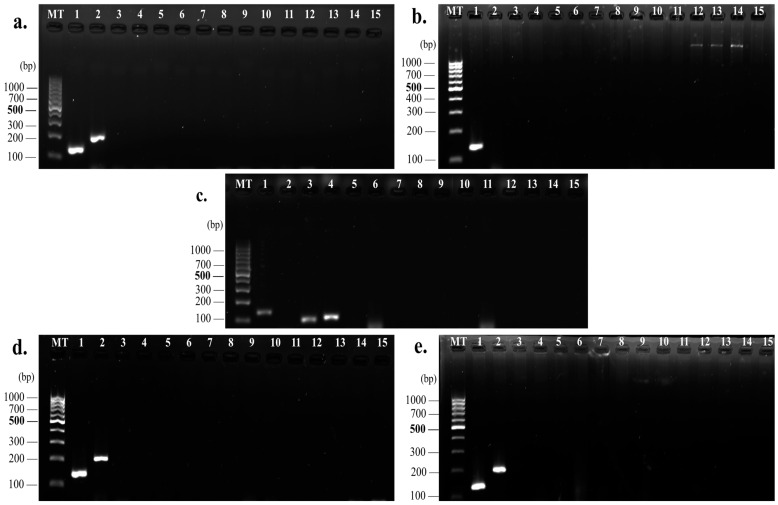
Visualization of PCR amplicons on a 2% agarose gel. (**a**) HFM 1; (**b**) HFM 4; (**c**) HFM 7; (**d**) HFM 11; (**e**) HFM 12. MT: DNA size marker (GeneRuler 100 bp DNA Ladder). Lane details: 1—*ent*A; 2—*entB*; 3—*entL50A*; 4—*entL50B*; 5—*entP*; 6—*entQ*; 7—*entAS-48*; 8—*ent31*; 9—*cyl*; 10—*entCRL35*; 11—*munKS*; 12—*esp*; 13—*gelE*; 14—*vanA*; 15—*vanB*.

The genetic screening demonstrated diverse enterocin profiles among the strains. The *entA* gene was ubiquitously present (100%, 5/5 strains), while *entB* was detected in three strains (60%, 3/5). The *entL50A/B* genes were exclusively harbored by strain HFM7 (20%, 1/5) (Figure 9). The antimicrobial activity patterns strongly correlated with the enterocin genetic profiles. Strain HFM7, carrying *entA*, *entL50A*, and *entL50B*, exhibited the highest inhibitory activity (20.0 ± 1.0 mm), suggesting potential synergistic effects among the encoded bacteriocins. Strains harboring both *entA* and *entB* (HFM1, HFM11, and HFM12) displayed substantial inhibition zones of 17.7 ± 1.5 mm, 18.7 ± 2.1 mm, and 16.3 ± 1.2 mm, respectively (Figure 10a,d,e). Notably, HFM4, which carried only *entA*, demonstrated significant antimicrobial activity (17.3 ± 1.5 mm) (Figure 10b), highlighting the potency of enterocin A.

The molecular characterization revealed variable enterocin expression patterns among the analyzed strains. Notably, strains HFM6, HFM9, and HFM10, while harboring the *entA* gene, displayed no detectable inhibitory activity, indicating potential regulatory or post-transcriptional mechanisms affecting bacteriocin production. Safety screening demonstrated the absence of key virulence determinants (*esp*, *gel*E, *hyl*) and antibiotic resistance markers (*vanA, vanB, ermA, ermB, and aac(6′)-Ie-alph(2″*).

Consistent results were observed across strains HFM1, HFM11, and HFM12, which showed the detection of *ent*A and *ent*B at expected fragment sizes of 134 bp and 211 bp, respectively (Figure 10a,d,e). Similarly, HFM4 carried only *ent*A (138 bp) (Figure 10b), while HFM7 displayed *ent*A (138 bp), *ent*L50A (135 bp), and *ent*L50B (135 bp), indicating distinct genetic profiles (Figure 10c). These profiles corresponded with the genetic diversity identified during genotyping.

The proteinaceous nature of the antimicrobial compounds was confirmed by the complete loss of inhibitory activity following trypsin treatment, supporting the identified enterocin genes. The strong correlation between genetic profiles and antimicrobial activity, particularly in multi-enterocin producers like HFM7, highlights their potential as effective biopreservative agents. This genetic diversity and robust antimicrobial potential position these *E. faecium* strains as promising candidates for clean-label and sustainable food preservation methods.

## 4. Discussion

This study comprehensively identified and characterized *E. faecium* strains isolated from dried figs marinated in olive oil. This traditional food, renowned for its biological benefits, has recently gained attention for its potential effects on Alzheimer’s disease [63] and its antimicrobial properties [64,65]. These benefits are primarily attributed to its richness in probiotics and the diversity of strains, particularly *E. faecium* [28].

The validation of MALDI-TOF MS identification through 16S rRNA gene sequencing reinforces the accuracy and reliability of the initial classification of the isolates as *E. faecium*. The successful amplification of ~1500 bp fragments in all isolates and their clear visualization on agarose gel (Figure 2) confirm the integrity and specificity of the colony-PCR process. This method, employed as a rapid, cost-effective, and reliable alternative to conventional DNA extraction techniques, eliminates the need for time-consuming and expensive DNA purification steps while maintaining high specificity and efficiency. By directly using isolated and pure bacterial colonies as a template, colony-PCR enables faster processing and high-throughput screening, making it highly suitable for microbial identification workflows [66].

Moreover, the high sequence similarity (>99%) with *E. faecium* reference strains in the NCBI GenBank database further supports the accuracy of the MALDI-TOF MS results. These findings highlight MALDI-TOF MS as a highly reliable and rapid identification tool, capable of differentiating bacterial species with high efficiency [67]. Compared to conventional molecular techniques, MALDI-TOF MS offers a significant advantage in terms of speed, cost-effectiveness, and ease of use, making it a powerful alternative for routine microbial identification [68,69].

The spectral profiles obtained for specific *E. faecium* strains revealed intermediate phenotypic diversity (Figure 1). Conserved biomarkers were observed in strains such as HFM4, HFM5, and HFM8, whereas variations in protein expression were detected in others, including HFM3, HFM6, and HFM7. Such differences may reflect underlying genetic or metabolic variability influenced by factors such as the origin of the culture medium or specific environmental adaptations. Notably, variations in the 9000–10,000 Da range could indicate differences in antimicrobial resistance mechanisms or genetic diversity among strains.

The protein spectra obtained align with previously reported profiles for *E. faecium*, reinforcing their utility for strain identification in both food safety and medical applications. Strains exhibiting unique biomarkers in the higher molecular weight range (9000–10,000 Da) may serve as valuable targets for further investigation into antimicrobial resistance or genetic diversity. Additionally, variations observed in the 5000–8000 Da range are consistent with bacteriocin production, as supported by prior studies on enterocin-producing *E. faecium*. For instance, bacteriocins such as enterocin IT (6390 Da) [70] and enterocins L50A and L50B (approximately 5203 and 5215 Da) have been identified in *E. faecium* [71]. These findings suggest that inter-strain variability offers opportunities to screen for strains with specific antimicrobial properties.

Genotypic analysis revealed substantial genetic heterogeneity among the isolates, with BOX-PCR demonstrating superior discriminatory power compared to ERIC-PCR and (GTG)5-PCR. This method’s ability to amplify less-conserved genomic regions allowed for fine-scale resolution of microbial diversity [72], particularly in the unique ecological niche of marinated dried figs [15]. The distinct clustering patterns observed in dendrogram analyses further underscored the evolutionary pressures shaping these strains, reflecting their adaptation to specific food environments. These results are consistent with prior studies documenting the genetic diversity of *E. faecium* in food matrices [48]. ERIC-PCR, despite attempts at optimization, exhibited limited resolution due to variability in the distribution of intergenic repetitive sequences. This finding aligns with earlier reports [49], further highlighting the need to select genotypic tools that match the specific ecological and taxonomic contexts of the isolates.

The results of this study underline the significant antimicrobial activity exhibited by *E. faecium* strains against the indicator strain *E. faecium* VCY, with evidence pointing to the production of bacteriocin-like inhibitory substances (BLIS). These findings align with prior research documenting the antimicrobial potential of *E. faecium*-derived bacteriocins. Almeida-Santos et al. [73] reported that a bacteriocin produced by *E. faecium* EFM01 exhibited inhibitory activity against other *E. faecium* strains. These studies, along with the present findings, highlight the versatility of *E. faecium*-produced bacteriocins in targeting diverse bacterial species, including closely related strains.

The tested *E. faecium* strains exhibited significant antimicrobial potential, particularly against Gram-positive bacteria, suggesting the production of bacteriocins, notably enterocins [74,75]. The pronounced inhibition of *M. luteus* GPE 3002 (17–21 mm), especially by HFM7 (20 ± 1 mm), confirms their efficacy, consistent with their mode of action targeting the bacterial membrane [76]. A synergistic antimicrobial effect between HFM1 and HFM4 against *M. luteus* GPE 3002 suggests the co-expression of enterocins A and B, which are often detected together and known for their combined action against various Gram-positive bacteria and lactic acid bacteria (LAB) [77,78,79]. This is further supported by our screening results (Figure 8).

Regarding Gram-negative bacteria, which are generally more resistant to bacteriocins due to their outer membrane rich in lipopolysaccharides, some *E. faecium* strains nevertheless inhibited *P. aeruginosa* ATCC 27853 (13–17 mm), with HFM7 showing the strongest inhibition (17 mm), possibly due to more efficient bacteriocin production. Unlike a previous study that reported no activity against *S. aureus* ATCC 25923 with an *E. faecium* strain, our results reveal moderate inhibition (12–15 mm). In contrast, HFM12 showed no activity, likely due to insufficient bacteriocin production or a restricted spectrum of action [80]. Only HFM4 and HFM7 inhibited *A. lwoffii* GPE 3002 (15–16 mm), while the other strains remained inactive, suggesting insufficient bacteriocin production or lower susceptibility of this bacterium. Previous studies also reported partial inhibition of Gram-negative bacteria by certain enterocins, indicating a potentially broader spectrum of action under specific conditions [2].

The effectiveness of bacteriocins depends on several factors. Their structure and nature determine their spectrum of action, with some enterocins being specific to Gram-positive bacteria, while others target a broader range. Their concentration plays a crucial role, as insufficient production may explain the lack of activity against certain pathogens [81]. Additionally, environmental parameters such as pH, temperature, and culture medium composition influence their efficacy [82]. *E. faecium* enterocins have been studied for their potential against clinical and foodborne pathogens. Furlaneto et al. [83] reported strong activity against *Listeria monocytogenes* and *S. aureus*, while Strateva et al. [84] demonstrated their ability to inhibit multidrug-resistant strains of *P. aeruginosa* and *Acinetobacter*. These findings confirm their potential for food biopreservation and combating multidrug-resistant pathogens, paving the way for further studies to better understand their mechanisms of action and optimize their production.

The variability in antimicrobial potency observed among the tested strains in this study further emphasizes the diversity of bacteriocin production and activity within bacterial populations. Such variability may stem from differences in genetic regulation, structural diversity of bacteriocins, or environmental factors influencing their expression. The absence of detectable activity in some strains under standardized conditions suggests that bacteriocin production may be strain-specific or triggered by specific environmental cues. The detection of key enterocin genes, *entA*, *entB*, *entL50A*, and *entL50B*, highlights the strong antimicrobial potential of the isolates. The universal presence of the *entA*-encoding gene occurred at high frequency among the tested *E. faecium* strains, which is similar to the rate found in previous studies [77], as it has been widely associated with significant activity against foodborne pathogens such as *L. monocytogenes* [24,85]. The co-occurrence of *entA* and *entB* in isolates such as HFM1, HFM11, and HFM12 suggests synergistic interactions that enhance antimicrobial efficacy [86]. This synergism has been reported in similar studies focusing on bacteriocin-producing LAB in fermented foods [24]. *entB* is possibly secreted by the dedicated transport proteins of *entA*, which is the reason why the *entB* gene is always associated with the *entA* gene, as found by Ogaki et al. [87].

The detection of the *ent*L50*A* and *entL50B* genes in this HFM7 strain highlights its antimicrobial potential [85], as demonstrated by the significant inhibition zone observed against *E. faecium* VCY (20.0 ± 1.0 mm, *p* < 0.001) among the tested strains. The simultaneous presence of *entL50A/B* and *ent*A in the same strain is low in frequency. For example, in a previous study by Ogaki et al. [46] on 135 enterococci strains, the *entL50A* and *entL50B* genes were not detected. It should be noted that the frequency of a single enterocin gene was higher than that of multiple enterocin gene combinations. Other researchers have shown that the presence of more than two genes in the same strain is due to the ecological niche of food sources specifically [82,87,88]. This uniqueness underscores the exceptional nature and potential of HFM7, as well as the importance of strain-specific effects and the niche-specific distribution of these genes. Enterocin L50A/B consists of two peptides, L50A and L50B, which synergistically promote their antimicrobial activity. They encode leaderless bacteriocins that exhibit a broad spectrum of antimicrobial activities against foodborne pathogens, including *L. monocytogenes*, *S. aureus*, *Bacillus cereus*, *Clostridium botulinum*, *Streptococcus pneumoniae*, *Streptococcus mitis*, *Streptococcus oralis*, *Streptococcus parasanguis*, *Streptococcus agalactiae*, and *Clostridium perfringens* [89]. Previous studies have demonstrated similar findings in artisanal dairy products, where strains harboring these genes displayed strong biopreservative potential [87,90]. Such findings are particularly significant in the context of clean-label food production, as these isolates offer a natural solution to controlling spoilage and pathogenic microorganisms.

The absence of virulence genes and antibiotic resistance genes (*vanA*, *vanB*, *ermA*, *ermB*, and *aac(6′)-Ie-alph(2″)* across all isolates reinforces their safety for food applications. This is critical given the dual nature of *Enterococcus* species, which can act as both beneficial probiotics and opportunistic pathogens [16,91,92]. The absence of undesirable genetic traits enhances the applicability of these isolates in the food industry, particularly in the development of starter cultures and biopreservatives.

Consistent with earlier studies [93,94], the isolates in this study exhibited strong antimicrobial potential without compromising safety. This positions them as ideal candidates for natural food preservation, aligning with the global demand for sustainable and minimally processed food systems. The findings of this study underscore the potential of integrating traditional food ecosystems into modern biotechnological frameworks.

Dried figs marinated in olive oil represent a culturally significant and nutrient-rich preparation, providing a novel resource for isolating antimicrobial-producing LAB. The universal presence of *entA* and the absence of virulence and resistance genes strengthen the suitability of these strains for clean-label food production. Such applications not only enhance food safety and shelf life but also cater to consumer preferences for natural and sustainable preservation techniques [8,12,95].

## 5. Conclusions

This study provides valuable insights into enterocin-producing *E. faecium* strains isolated from dried figs marinated in olive oil, highlighting their potential as natural food preservatives. Genotypic analyses, particularly BOX-PCR, revealed significant genetic diversity among the isolates, while functional screening confirmed robust antimicrobial activity associated with the detection of the *ent*A, *ent*B, and *ent*L50A/B genes. The genotype-phenotype correlation analysis identified strain HFM7 as the most potent antimicrobial strain, exhibiting the highest inhibition zone (20.0 ± 1.0 mm) and harboring three enterocin genes (*ent*A, *ent*L50A, and *ent*L50B). The antimicrobial potential of *E. faecium* strains, particularly against Gram-positive and select Gram-negative pathogens—along with their lack of virulence and antibiotic resistance genes—underscores their promise as natural antimicrobial agents for food preservation. Usually, bacteriocins from Gram-positive bacteria express greater activity than those from other Gram-positive bacterial species. Moreover, it was suggested that in some cases, the combined application of bacteriocins and other antimicrobials can express synergistic interactions and improve the antimicrobial performance of the bacteriocins from Gram-positive bacteria, including versus some Gram-negative species, a topic that deserves further attention and investigation. These findings emphasize the importance of integrating molecular and functional analyses for precise strain characterization and highlight the value of traditional food ecosystems as reservoirs of bioactive microorganisms. By bridging cultural heritage and modern food biotechnology, this study supports the development of sustainable, clean-label preservation strategies that align with consumer preferences for natural and minimally processed foods. While these results establish the antimicrobial potential and safety of *E. faecium* strains, further research is necessary to explore their scalability for industrial applications. Additionally, comprehensive regulatory evaluations will be essential to ensure compliance with safety standards for commercial use.

## Data Availability

The data supporting the findings of this study are available from the corresponding author upon reasonable request.
